# PTSD and Suicidal Behaviors Amongst L'Aquila 2009 Earthquake Young Survivors

**DOI:** 10.3389/fpubh.2021.590753

**Published:** 2021-02-10

**Authors:** Claudia Carmassi, Carlo Antonio Bertelloni, Valerio Dell'Oste, Chiara Luperini, Donatella Marazziti, Rodolfo Rossi, Liliana Dell'Osso

**Affiliations:** ^1^Department of Clinical and Experimental Medicine, Section of Psychiatry, University of Pisa, Pisa, Italy; ^2^Department of Experimental Medicine, University of L'Aquila, L'Aquila, Italy; ^3^Department of Systems Medicine, University of Rome Tor Vergata, Rome, Italy

**Keywords:** natural disasters, earthquakes, post-traumatic stress disorder, suicidality, suicide attempts, suicidal ideation, trauma and loss spectrum-self report, mood spectrum-self-report

## Abstract

**Background:** Post-traumatic stress disorder (PTSD) is one of the most frequent and severe psychiatric consequences of natural disasters, frequently associated with suicidality. The aim of this study was at examining the possible relationships between suicidal behaviors and full-blown or partial PTSD, in a sample of young earthquake survivors. The second aim was at investigating the specific role of PTSD symptoms on suicidality.

**Methods:** A total of 475 young adults who survived the L'Aquila 2009 earthquake, one of the most severe Italian disasters of the last decades, were recruited and assessed after 21 months from the catastrophe. Participants were evaluated by two questionnaires assessing subthreshold psychopathology, the *Trauma and Loss Spectrum Self-Report* (TALS-SR) to investigate both full and partial PTSD, and two specific *Mood Spectrum Self-Report* (MOODS-SR) sub-domains exploring suicidality, namely *suicidal ideation* and *suicide attempts*.

**Results:** The ensuing findings showed that suicidal ideation and suicide attempts were present, respectively, in 40 (8.4%) and 11 (2.3%) survivors. Rates of suicidal ideation were significantly more elevated in full-blown PTSD subjects (group 1), as compared with those suffering from partial (group 2) or no PTSD (group 3). Interestingly, group 2 subjects showed significantly more suicidal ideation than healthy individuals, and less than those of group 1, while the frequency of suicide attempts was similar across the three groups. Suicidal ideation was associated with higher scores in the following TALS-SR domains: *grief-reactions, re-experiencing, avoidance and numbing, maladaptive coping*, and *personal characteristics/risk factor*.

**Conclusions:** The results of the present study support and extend previous findings on the role of PTSD symptoms in suicidality after a severe earthquake. However, as compared with available literature, they also highlight the significant impact of sub-threshold PTSD manifestations in increasing the suicide risk in survivors of a mass disaster.

## Introduction

Earthquakes are one of the most frequent and disruptive natural disasters, as they expose involved population to not only injuries, devastation and death, but also severe psychological and psychiatric consequences. Indeed, during the past 40 years, a six-fold increase in the number of earthquakes worldwide has been reported, leading to an increased incidence and prevalence of different psychiatric disorders amongst survivors (1). Post-traumatic stress disorder (PTSD) is the most frequent psychiatric sequela following earthquakes, with prevalence rates ranging between 11.7 and 82.6% amongst survivors, depending on the characteristics of the population involved, the diagnostic criteria used to assess diagnosis and the traumatic event intensity (2–8). Suicidal behavior is not an uncommon consequence of earthquakes (9, 10), so that, not surprisingly, these natural disasters are widely investigating to assess their possible impact on this dramatic phenomenon (11). A great amount of data show increased rates of suicidal ideation and attempts, as well as of completed suicide, especially after highly destructive earthquakes (12–14). Again, PTSD seems to be one of the main factors linking a mass trauma to suicidality (15–19). A significant association between PTSD symptoms and suicide ideation emerged in about 2,300 child and adolescent survivors of the Wenchuan earthquake in China (17). Another study showed increased rates of PTSD and suicidal attempts, rather than depressive, or anxiety disorders, in a cohort of Swedish tsunami survivors, while suggesting that the effect of the trauma on suicidality was largely depending on stress-related disorders (18). The risk for suicidal behaviors, including suicidal ideations, plans, and attempts, in 1,369 adults exposed to an earthquake resulted to be about 11%, with suicide attempts being related to the presence of PTSD (19).

L'Aquila 2009 earthquake, that occurred on April 6, 2009, is so called for its proximity to L'Aquila, the main town in the Abruzzi region of central Italy. The magnitude 6.3 tremor struck at 3:32 a.m. local time, damaging extensively the 13th-century town, located only about 60 miles (100 km) northeast from Rome. As a whole, 309 people died, more than 1,600 were injured and approximately 66,000 were displaced to temporary settlements (9). Previous studies of our group on different samples of survivors from the L'Aquila 2009 earthquake, showed suicidality to be related to PTSD diagnosis, with rates of suicidal ideation and attempts ranging, respectively, between 7 and 12%, and between 0.8 and 14% (4, 20–22). Furthermore, some authors have criticized the current diagnostic criteria for PTSD, considering them too restrictive. The diagnostic and statistic manual for mental disorders (DSM-5) criteria for PTSD (23) diagnosis encompass the endorsement of four symptom clusters related to the traumatic event, particularly intrusion symptoms (criterion B), avoidance (criterion C), negative alterations in cognitions and mood (criterion D), and alteration in arousal (criterion E). These diagnostic procedure could fail to include a large number of victims of traumatic events (3, 4, 24, 25). Indeed, despite only a few people exposed to a trauma suffer from a full-blow PTSD that can be easily recognized and treated, other individuals may develop PTSD symptoms classified as partial or subthreshold PTSD (26–28). This subthreshold psychopathology generally leads to a significant impairment, and it may require intervention and treatment (29–31). Various definition of partial PTSD have been reported in literature, so it could be difficult to compare findings from different studies. Accordingly, the prevalence of partial PTSD in earthquake survivors seems to range between 19 and 29% in the different studies (3, 4), possibly depending on the heterogeneous conceptualizations and diagnostic assessments. In the 2015, within the framework of the world health organization *World Mental Health Surveys*, some authors proposed a new definition for partial PTSD, according to the DSM-5 (23). McLaughlin et al. (27) proposed to define the presence of partial PTSD as the endorsement of two or three DSM-5 criteria B-E. Partial PTSD, similar to full-blown PTSD, has been related to increased suicidal behaviors, amongst earthquake survivors (3). Further, a comparable risk of suicide ideation in both full-blow and partial PTSD was also reported in a sample of 275 war veterans, while suggesting the importance to evaluate and to treat subthreshold trauma-related psychopathology (32). However, to the best of our knowledge, no studies utilized the new proposed criteria for partial PTSD in a sample of survivors from a natural disaster, such as an earthquake, or investigated their association with suicidality. Therefore, the present study was aimed at examining the possible relationships between suicide behaviors and either full-blow or partial PTSD, according to DSM-5 criteria, in a sample of L'Aquila 2009 earthquake survivors. The second aim was at investigating the specific role of PTSD symptoms on suicidality.

## Methods

### Study Participants

The sample included 512 subjects recruited amongst high-school senior students who had experienced the L'Aquila 2009 earthquake, 21 months earlier. All subjects were directly exposed to the disaster and received assistance in emergency conditions. Full data were available for 475 out of the total of 512 subjects (94.2% of the overall sample) (women: 203, 42.7%; men: 272, 57.3%; mean age 17.67 ± 0.78 years). Detailed characteristics of the study sample are reported elsewhere (4, 6).

The school council and the Ethics Committee of the University of L'Aquila approved all recruitment and assessment procedures. Eligible subjects or their legal representatives (for underage individuals) provided written informed consent to participate in the study that given was carried out in accordance with the declaration of Helsinki.

### Instruments and Assessments

Participants were evaluated by two specific questionnaires, the so-called “Trauma and Loss Spectrum–Self Report (TALS-SR)” (33) to examine post-traumatic stress symptoms, and by a subscale of the “Mood Spectrum–Self Report (MOODS-SR)” (34) to investigate suicidal behaviors.

The TALS-SR encompasses 116 items, organized in nine domains, exploring the lifetime experience of a range of loss and/or traumatic events and lifetime symptoms, behaviors, and personal characteristics that could represent manifestations and/or risk factors for the development of a trauma or stressor related disorders. The nine domains explored by the TALS-SR are: *loss events* (I); *grief reactions* (II); *potentially traumatic events* (III); *reactions to losses or upsetting events* (IV); *re-experiencing* (V); *avoidance and numbing* (VI); *maladaptive coping* (VII); *arousal* (VIII); and *personal characteristics/risk factors* (IX). Items responses are coded in a dichotomous way (yes/no) and domain scores are obtained by counting the number of positive answers. To accomplish the aims of the present study, the instrument was adapted to examine symptoms related to L'Aquila 2009 earthquake. According to previous studies (6, 35), the presence of PTSD was assessed by means of TALS-SR items corresponding to DSM-5 criteria for PTSD diagnosis. Specifically, we utilized the following matching between symptom criteria and TALS-SR items:

Criterion B (B1 = 80; B2 = 77; B3 = 79; B4 = 78; B5 = 81);

Criterion C (C1 = 86; C2 = 87; and/or 88 and/or 89);

Criterion D (D1 = 90; D2 = 95; D3 = 85; D4 = 96; D5 = 91; D6 = 93; D7 = 92);

Criterion E (E1 = 108; E2 = 99 and/or 100 and/or 102 and/or 103 and/or 104; E3 = 106; E4 = 107; E5 = 105; E6 = 109).

To assess the presence of partial PTSD, we adopted the criteria proposed by previous studies corresponding to the endorsement of two or three symptom clusters (27, 36).

The MOODS-SR, a questionnaire exploring mood spectrum symptoms, includes 161 items coded as present/absent, for one or more periods of at least 3–5 days after the earthquake exposure. The items are organized into manic and depressive components as well as into a section that assesses disturbances in rhythmicity and vegetative functions, yielding a total of seven domains. In fact, both the manic and the depressive components are subtyped into three domains exploring mood, energy, and cognition symptoms, respectively. Suicidality was evaluated by means of six specific items of this questionnaire exploring both suicidal ideation and attempts after the earthquake exposure. The six MOODS-SR items combined to assess the suicidality are as it follows: “*thought that life is not worth living*” (item 102); “*wished he/ she would not wake up in the morning, or he/she would die in an accident or from something like a heart attack or a stroke*” (item 103); “*wanted to die or hurt him/herself* ” (item 104); “*wanted to die and had a specific plan to hurt or kill him/herself* ” (item 105); “*actually committed a suicide attempt*” (item 106); and “*committee a suicide attempt that required medical attention*” (item 107). The first four items were combined in order to assess a new dichotomous variable named *suicidal ideation* that resulted to be endorsed when at least one of the items was endorsed. A second new dichotomous variable named *suicidal attempts* was derived from the endorsement of at least one out of the last two items (106 and/or 107).

### Statistical Analysis

The Chi-square analysis was used to compare rates of *suicidal ideation* and *suicidal attempts* between subjects with PTSD, partial PTSD and without PTSD. Chi-square test was computed also to compare rates of *suicidal ideation* and *suicidal attempts*, between male survivors and female ones. In the subgroup of survivors with full or partial PTSD, the *t*-test was carried out to evaluate differences in TALS-SR domain scores between those with and without suicidal ideation. All statistical analyses were carried out using the Statistical Package for Social Science (SPSS Inc., Chicago 2006), version 22.0.

## Results

One hundred sixty-nine (35.6%) and 202 (42.5%) earthquake survivors were suffering from respectively, full and partial PTSD, according to DSM-5 criteria.

**Figure 1:**
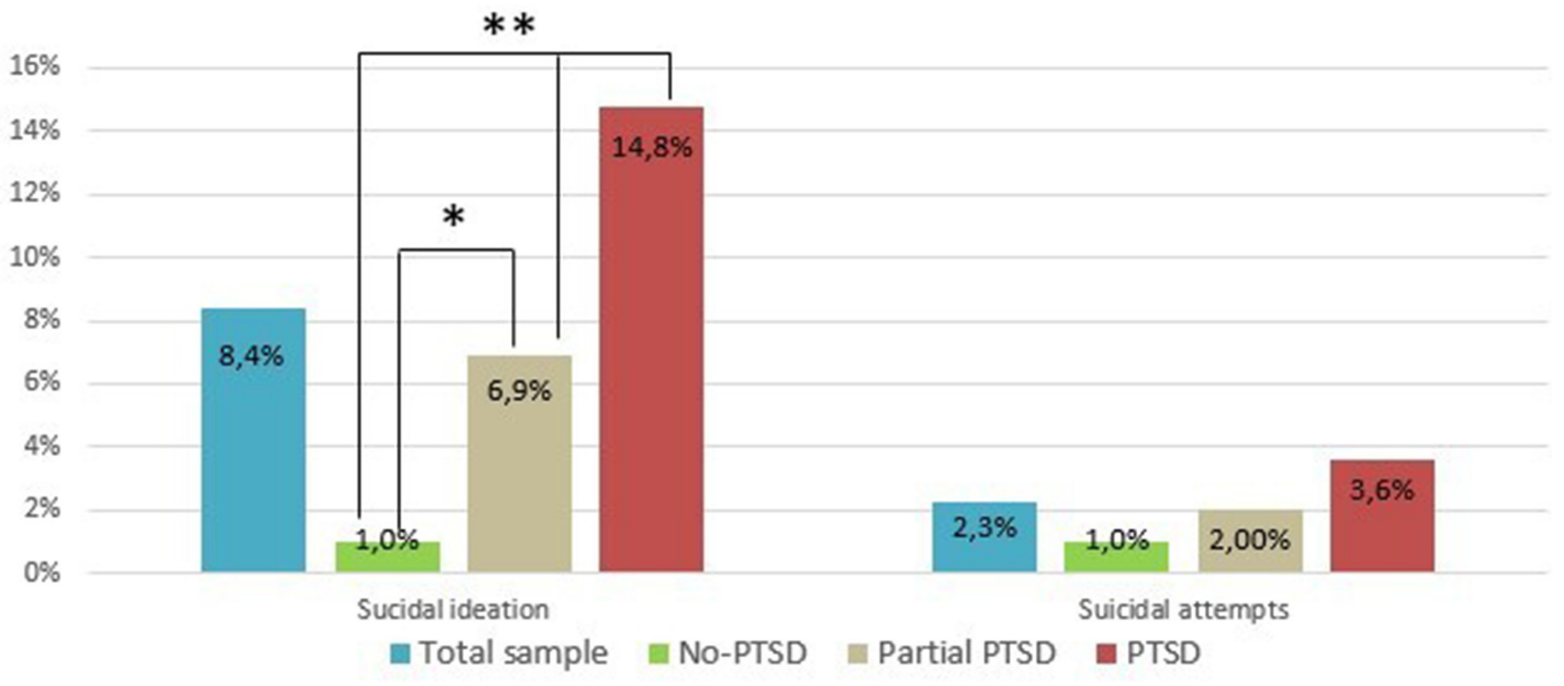
Suicidal ideation and suicidal attempts rates in the total sample (*N* = 475), No-PTSD (*N* = 104), partial PTSD (*N* = 202), and PTSD (*N* = 169) groups. *significantly higher rates respect to No-PTSD (*p* = 0.022). **significantly higher rates respect to partial PTSD (*p* = 0.014) and No-PTSD (*p* < 0.001).

Rates of suicidal ideation and suicidal attempts in the total sample were, respectively, 8.4% (*N* = 40) and 2.3% (*N* = 11) (Figure 1).

Subjects with full-blown PTSD showed significantly higher *suicidal ideation* (25, 14.8%) than those with partial PTSD (14, 6.9%; *p* = 0.014) or those without PTSD (1, 1%; *p* < 0.001). Further, participants with partial PTSD showed significantly higher suicidal ideation than those without PTSD (*p* = 0.022). No statistically significant intergroup differences were found in *suicidal attempts* across the three groups. Rates of *suicidal ideation* were not statistically different between males and females [18 (6.6%) vs. 22 (10.8%), *p* = 0.141] in the total sample. No gender differences emerged also for *suicidal attempts* [8 (2.9%) vs. 3 (1.5%), *p* = 0.459].

In survivors with full or partial PTSD, the TALS-SR domains *grief-reactions, re-experiencing, avoidance and numbing, maladaptive coping*, and *personal characteristics/risk factors* scores were significantly more elevated in those subjects presenting *suicidal ideation*, as compared with those without it (Table 1).

**Table 1:** TALS-SR domain scores in survivors with or without suicidal ideation amongst subjects with full or partial PTSD.

**TALS-SR domains**	**Total (*N* = 371)**	**No suicidal ideation (*N* = 332)**	**Suicidal ideation (*N* = 39)**	***p***
(I) Loss events	3.12 ± 1.63	3.09 ± 1.62	3.41 ± 1.66	0.247
(II) Grief-reactions	10.13 ± 5.32	9.81 ± 5.24	12.82 ± 5.32	0.001
(III) Potential traumatic events	3.44 ± 2.45	3.32 ± 2.29	4.43 ± 3.42	0.055
(IV) Reactions to losses or traumatic events	8.12 ± 2.99	8.02 ± 2.93	8.97 ± 3.36	0.059
(V) Re-experiencing	4.02 ± 2.04	3.94 ± 1.99	5.26 ± 2.28	0.037
(VI) Avoidance and numbing	4.14 ± 2.28	4.01 ± 2.25	5.88 ± 1.69	0.001
(VII) Maladaptive coping	1.04 ± 1.39	0.89 ± 1.24	2.28 ± 1.86	<0.001
(VIII) Arousal	2.60 ± 1.44	2.55 ± 1.45	2.97 ± 1.27	0.088
(IX) Personal characteristics/risk factors	2.73 ± 1.60	2.66 ± 1.58	3.33 ± 1.61	0.013

## Discussion

To the best of our knowledge, this is the first study examining the relationship between DSM-5 full and partial PTSD and suicidal behavior in young survivors of the severe earthquake that occurred in 2009 in L'Aquila, the main town of a region from central Italy.

The ensuing findings revealed that the high-school senior students included in the present study showed suicidal ideation and suicidal attempt rates of, respectively, 8.4 and 2.3%. Our results are in agreement with previous data on earthquake survivors reporting suicidal thoughts in around 5–19% of subjects depending on the sampling selection and the severity of the catastrophe (37–41). Vehid et al. described 16.8% of suicidal ideations in 3,609 adolescents after 2 months from the Marmara earthquake. Another study detected a prevalence of suicidal ideation of 10.6% amongst 3,324 Chinese secondary school students exposed 1 month earlier to the Sichuan earthquake (38). On the contrary, other studies noted lower rates (0.4 and 3%), although it should be underlined that suicidal behaviors were rarely assessed amongst adolescents and young adults exposed to natural disasters (42–44).

As expected, the analysis of the intergroup differences showed that survivors with DSM-5 partial PTSD had intermediate levels of suicidal ideation, that were significantly different in comparison with both those with or without PTSD. Our results strongly support the notion that an exposure to a natural disaster, particularly to a high disruptive earthquake can eventually increase the risk for suicide behaviors and possibly promote a negative outcome in young subjects (11).

It is important to recall that we assessed survivors almost 2 years after the earthquake, so that this elapsed time might have also influenced the suicidality rates reported. In this regard, epidemiologic surveys conducted after 2 years from this event noted decreased rates of death by suicide with respect to the 5 years before the earthquake (45, 46). This is in line with previous research highlighting a reduction of the number of suicides in the 2 years post-disaster period. Conversely, we reported an increased number of suicides from the 3rd year after the catastrophe, during the “disillusionment” phase. This latter represents the period after 1 year or more after the disaster, when mental health problems can occur, also because of the disappointment and frustration toward the slow pace of resource allocation and reconstruction (45).

Taken together, our findings corroborate and extend the results of previous studies highlighting the role of PTSD as a relevant risk factor for suicidal ideation (47–50). In military veterans, PTSD diagnosis was associated to a six-fold increased risk of suicidal thought (51), and this relationship was also confirmed in civilian samples (49, 50, 52–54). Furthermore, PTSD individuals also resulted at higher risk for suicide attempts, especially if young or male (16, 54–57). On the contrary, no sex-related differences were noted in our sample. As far as earthquake survivors are concerned, recent data pointed out an association between PTSD and suicidal attempts in 1,369 survivors 8 years after the disaster (41).

In addition to available findings, our study underlined a strong relationship between partial DSM-5 PTSD and suicide ideation. Previously, subthreshold PTSD symptoms were already associated with current suicidal ideation in a national survey on 9,358 subjects (58). This study was followed by another one on 252 earthquake survivors supporting the impact of partial PTSD on suicidality (3). A more recent study on 1,484 veterans described a relationship between DSM-5 partial PTSD and lifetime or current suicidal ideation (36). Our results strongly support the association between DSM-5 subthreshold PTSD symptoms and suicidal ideation, while confirming the relationship even in a civilian sample exposed to a mass trauma. On the contrary, suicidal attempts were not significantly related to full or partial PTSD in our sample, maybe for the small sample size and its relatively homogeneity and similar life habits of the subjects included that were all high-school senior students. In any case, to the best of our knowledge, there is a lack of studies in the literature about the role of subthreshold PTSD manifestation on the risk of suicide attempts, so that we are of the opinion that further studies in larger samples are warranted.

Four symptomatic TALS-SR domains, specifically, *grief reactions, re-experiencing, avoidance and numbing*, and *maladaptive coping*, were significantly higher in subjects with full-blow or partial PTSD reporting suicidal ideation, respect to those without this ideation. The relationship between PTSD symptom clusters and suicidal ideation is a primary topic for researchers and clinicians, in order to screen patients at higher risk for suicide and to develop targeted treatment strategies (59). Bereavement and pathological grief manifestations were related to PTSD severity and suicidality (60). Our data, exploring by the TALS-SR if the subjects had reported the loss of a close friend or relative during the earthquake that had caused the death of about 306 people in a little city, confirmed previous findings on the impact of a loss event on the post-traumatic stress reactions followed a mass trauma. Despite most of the previous studies on PTSD according to DSM-5 criteria showed an association between suicidal ideation and symptoms of hyperarousal, anhedonia, or negative affect (61–63), other researches reported contradictory results. A recent study also detected a relationship between suicidality and intrusive symptoms and avoidance, while presenting no hierarchy amongst PTSD symptom clusters on their specific impact on suicidal ideation (59). Furthermore, also externalizing manifestations, such as reckless or self-destructive behavior, alcohol or drug abuse and self-injuring are frequently observed in subjects with PTSD (16, 20, 62, 64). These symptoms, encompassed in the TALS-SR *maladaptive coping* domain, were related to suicidal ideation in our sample.

Several limitations of the study should be kept in mind when interpreting our results. As already mentioned, the first is the small sample size, although we attempted to recruit all existing high schools exposed to the earthquake. The second limitation is the use of self-report instruments, instead of clinical assessment that may be considered less accurate, and of no standardized and validated scale to assess suicide-related behaviors. The third is the lack of information about social support or Axis I psychiatric comorbidities that could affect suicidality, such as mood disorders (22, 52–54, 65). Furthermore, data about pre-existent psychiatric comorbidity and previous traumatic events are not available in our study. These variables, in fact, could influence peri-traumatic reactions during the earthquake or PTSD severity. Finally, the homogeneity of the study sample that included only non-clinical high school students and the cross sectional design of the study that did not allow to explore eventual changes in suicidality rates according to time after the exposure. However, it is also true that the homogeneity of the sample might also be a strength of the study.

In conclusion, our study indicates that both full and partial PTSD young subjects might be at risk for suicidal ideation, indicating the importance of evaluating and possibly treating even the subthreshold post-traumatic stress manifestations in survivors of a natural disaster. Since several symptoms, such as grief manifestations, re-experiencing, active avoidance, numbing, and maladaptive behaviors were related to suicidal thoughts, they should represent primary targets for interventions in the framework of PTSD management.

## Data Availability Statement

The data supporting the findings of the article are not publicly available, but it can be provided by the corresponding author on reasonable request.

## Ethics Statement

The studies involving human participants were reviewed and approved by Ethics Committee of the University of L'Aquila. Written informed consent to participate in this study was provided by the participants' legal guardian/next of kin.

## Author Contributions

CC and LD participated to the conception and design of the study. CC, CB, and LD participated to the interpretation of the data, the draft, and critical revision of this article. CB undertook the statistical analysis. VD, CL, RR, and DM participated to the critical revision of the manuscript. All authors agreed to be cited as co-authors, accepting the order of authorship, and approved the final version of manuscript and the manuscript submission to Frontiers in Psychiatry.

## Conflict of Interest

The authors declare that the research was conducted in the absence of any commercial or financial relationships that could be construed as a potential conflict of interest.
